# Socioeconomic driving forces behind air polluting emissions in Mexico

**DOI:** 10.1371/journal.pone.0292752

**Published:** 2023-10-12

**Authors:** Mayra Vega-Campa, Francisco J. André, Mario Soliño

**Affiliations:** 1 Complutense University of Madrid, Madrid, Spain; 2 Department of Economic Analysis, Faculty of Economics, Complutense University of Madrid, Pozuelo de Alarcón, Madrid, Spain; 3 Complutense Institute for International Studies (ICEI), Finca Mas Ferré, Pozuelo de Alarcón, Madrid, Spain; 4 Institute of Marine Research—CSIC, Vigo, Spain; University of Agriculture Faisalabad, PAKISTAN

## Abstract

Air pollution is one of the most severe environmental problems that Mexico is currently facing. The objective of this paper is to quantify the most relevant socioeconomic driving forces behind air polluting emissions and, more specifically, 7 local pollutants in Mexico. We do so in a multilevel version of the Stochastic Impacts by Regression on Population, Affluence and Technology (STIRPAT) model that accounts for the spatial heterogeneity at the municipal level across the country. The results show that the most relevant variables to determine the emissions of atmospheric pollutants are the population, the harvested area and the number of cars, while technological development helps to mitigate such emissions. The ecological elasticities are, in all cases, smaller than one. Our purpose is to provide quantitative information about these socioeconomic driving forces of air deterioration as a basis to establish some recommendations for environmental policy decision-making.

## 1. Introduction

Air pollution is one of the greatest environmental risks to human health and ecosystems. Human activity generates large amounts of atmospheric pollutants, with negative effects at global and local scales. As it has been widely documented by the IPCC reports, greenhouse gases (GHGs) contribute to overheating the planet and, consequently, have important effects on a global scale. Fighting climate change has become, not only a long-term, but even a short-term challenge, since future scenarios predict unstoppable global warming if we do not immediately act. Carbon neutrality is one the challenges to the next decades, especially in some parts of the world [1]. Although the original objectives of mitigation policies for climate change and air pollution are nor related, the reduction of CO_2_ emissions has benefits on the local air pollution mitigation, and vice versa [2–4].

Therefore, along with global environmental problems such as climate change, there are other pollutants whose effects are mostly regional and local and also have significant negative effects on the well-being of society [5–7]. To a large extent, global and local polluting share common causes, such as the burning of fossil fuels (coal, oil and gas), mainly in the industrial processes, heating and road transport.

According to the World Health Organization [8], it is estimated that seven million people die each year from exposure to the fine particles contained in polluted air. These particles penetrate deep into the lungs and the cardiovascular system and cause diseases such as strokes, heart disease, lung cancer, chronic obstructive pneumopathy and respiratory infections, such as pneumonia.

While air pollution is a global problem, the poorest and developing nations bear the brunt as they tend to have weaker economies, less developed health and social systems and are thus less endowed to face the consequences. This article focuses on the case of Mexico, where poor air quality is seen as a paramount issue of concern, not only because air emissions contribute to global problems such as climate change, but also, for their domestic and local implications. As we explain below, we place the emphasis on the latter but addressing local rather than global pollutants.

The purpose of the study is twofold: (i) to identify and quantify the main socioeconomic driving forces of air deterioration in Mexico considering the spatial heterogeneity across the country, and (ii) to provide recommendations for environmental policy decision-making based on the quantitative results. For that, we use the Stochastic Impacts by Regression on Population, Affluence and Technology (STIRPAT) model [9–11]. In previous applications of the STIRPAT model, geographical heterogeneity along a specific country has not been considered. Therefore, our study contributes to the previous literature about the socioeconomic driving forces of air pollution in several ways. First, this study incorporates the geographical heterogeneity of emissions, using municipal rather than country-level data. Secondly, and consistent with this approach, we focus on local air pollutants rather than greenhouse effect gases. The pollutants most commonly addressed in the related literature are CO_2_ emissions (e.g. in Mexico, [12, 13]). From an econometric point of view, we use a multilevel model in order to exploit the structure of our data, which is nested in a two-level administrative structure because all the municipalities are grouped in a reduced number of regions or states.

This research is important to sustainability, carbon neutrality, local economy, environment, social development, etc. The paper is organized as follows. Section 2 provides a review of the analytical framework and the related literature; Section 3 presents the methodology and the data used in the study and Section 4 shows the results of the study. The fifth and last section provide the main conclusions and some policy suggestions.

## 2. Material and methods

### 2.1. Air pollution and human health in Mexico

The present study departs from most of the existing studies by focusing on 7 local air pollutants: suspended particles less than 10 micrometers (PM_10_)_,_ suspended particles less than 2.5 micrometers (PM_2.5_)_,_ sulfur dioxide (SO_2_), carbon monoxide (CO), nitrogen oxides (NO_X_), volatile organic compounds (COV), and ammonia (NH_3_)_._ These pollutants have in common that they are an important source of respiratory diseases, reduce visibility, contribute to acid rain formation and are precursors of smog and ozone.

One of the collateral effects of the development of both, global and local emissions mitigation policies, is the improvement of human health [4]. In fact, the most obvious dimension of the impact of air pollution, and the one that has been more widely studied in Mexico is the one related to human health [14–18]. It has been widely reported that atmospheric pollution can cause many diseases, especially respiratory ones. Exposure to air pollutants can cause coughing, respiratory tract irritation or decreased lung function, trigger asthma-like reactions, aggravate a previous asthma condition and increase cancer cases [19]. For example, according to the Global Burden of Disease [20], chronic exposure to PM_2.5_ has resulted in 7,600 premature deaths per year in Mexico, and recent evidence also reports that exposure to PM_2.5_ could increase the likelihood of COVID-19 death [14].

Air pollution also represents a serious economic problem due its associated costs, including the private expenses that people affected by various diseases must pay in medicines and medical consultations, as well as the consequences of decreasing their work or school productivity. In addition, the social costs of premature death materialize, on the one hand, in the loss of the contributions that these people could make to society and, on the other hand, in the psychological and emotional costs inflicted on family and friends. The increase in morbidity also provokes a loss of competitiveness due to absenteeism and the reduction in the hours dedicated to productive activities. Finally, the public resources, both human and material, used to care for people affected by air pollution can be interpreted as an opportunity cost, as such resources cannot be used to meet the other needs of the health sector [21].

### 2.2. Conceptual framework: The STIRPAT model

The IPAT identity, developed by [22], states that environmental impact can be split in three factors: the population size, the level of economic activity or per capita income and the polluting intensity of economic activity. The latter is fundamentally determined by the sectoral structure of the economy and the current production technology. In formal terms, this approach disaggregates environmental impact (I) as the product of the population size (P), wealth (A) and technology (T), i.e.:

I=P×A×T (1)

[9, 10] resumed this idea and proposed a stochastic version of the IPAT identity in order to analyze the impact of the population, wealth and technology on the CO_2_ emissions of 111 countries. In their approach the technological variable is modeled as a residual, i.e., as a non-observable term that encompasses all those factors that affect environmental impact and are neither population nor per capita economic activity. Subsequently, [23] revisited the IPAT identity and renamed the result as ImPACT. In the new version, the technology variable is not included in the error term, but is disaggregated in consumption per unit of product (C) and impact per unit of consumption (T), as follows:

I=P×A×C×T (2)

Based on the IPAT identity and the ImPACT model, [11] proposed a new model called STIRPAT. They pointed out that the previous approaches had a mere accounting nature, did not allow for hypothesis testing and assumed a linear specification. To overcome these limitations, STIRPAT is proposed as a non-linear stochastic model and incorporates the concept of ecological elasticity to measure the sensitivity of environmental impact to the anthropogenic forces included in the model. This approach also accounts for the technological variable T, which is assumed to represent all the factors that have an impact on environmental deterioration, other than population and per capita income. The STIRPAT model specification is:

$$I_{i}=aP_{i}^{b}\;A_{i}^{c}T_{i}^{d}e_{i}$$ (3)

where *I*, *P*, *A* and *T* have the same interpretation as in the IPAT identity and *e*_*i*_ is an error term. Subindex “*i*” refers to the different units (countries, regions, cities,) considered in the model. The parameters of the model are *a*, *b*, *c*, *d*, where *a* is a scale term and *b*, *c*, *d* are the ecological elasticities of environmental impact with respect to population, production and technology, respectively, which allow for inferences about the precise weight of this variables on environmental impact [11]. Thus, the STIRPAT model can be seen as a generalization of the IPAT identity or, in other words, the IPAT identity can be seen as a deterministic, particular case of the STIRPAT model with *a* = *b* = *c* = *d* = 1.

Applying natural logarithm on both sides of Eq (3) we get:

$$lnI_{i}=a_{0}+bln(P_{i})+cln(A_{i})+dln(T_{i})+u_{i}$$ (4)

$$\mathrm{where}\;a_{0}=lna\;\mathrm{and}\;u_{i}=lne_{i}.$$

Technological innovation and digital economy contribute to reduce emissions and achieve green development goals [24, 25]. The technological variable *T* can be disaggregated by including different factors that theoretically influence the environmental impact. Different variables have been used in the literature to proxy this variable. These include, among others, the energy efficiency or intensity [12, 13, 26–34], the economic weight of the industrial sector [11, 13, 26–28, 30, 33, 35–37], the share of the service sector in the GDP [13, 30, 36, 38], the level of trade openness [32], and the climatic conditions [11, 32].

### 2.3. The data

Our database includes data at the municipal level (2457 observations) for Mexico, which are grouped into 32 states. For the estimation of the models, we omitted incomplete observations (289), and ended up with 2168 effective observations. Table 1 shows the name of the variables, the code used for modelling using the statistical package STATA®, the units of measurement, data source, and descriptive statistics.

**Table 1:** Variables of the model.

Variable	Code	Unit of measurement	Data source	Obs.	Mean	Min.	Max.	Std. dev.
**Independent Variables**								
Population	POP	Number of people	State and Municipal Database System-INEGI	2457	48649	87	1827868	139060
Gross Production per capita	GPPC	Thousands of pesos per capita	State and Municipal Database System-INEGI	2457	43.50	0.027	9488.64	283.151
Households with access to computers and internet per capita	HCI	percentage	2015-INEGI Intercensal Survey	2298	0.024	<0.01	0.288	0.028
Harvested area	HA	Hectares	Agri-food and consultation system	2432	8513	6	227773	15557
Transport	TR	Number of cars (cargo and public) per thousand inhabitants	State and Municipal Database System-INEGI	2352	95.396	<0.01	1321.962	91.027
**Dependent Variables**								
Sulphur dioxide	SO_2_	Tons	National Emissions Inventory-SEMARNAT	2457	681	0.024	197,133	7,792
Carbon monoxide	CO	Tons	National Emissions Inventory-SEMARNAT	2457	3,123	11.318	98,635	7,335
Nitrogen oxides.	NO_X_	Tons	National Emissions Inventory-SEMARNAT	2457	1,569	7.862	58,746	3,785
Volatile organic components	COV	Tons	National Emissions Inventory-SEMARNAT	2457	5,805	19.970	493,707	18,099
Suspended particles less than 2.5 m	PM_2.5_	Tons	National Emissions Inventory-SEMARNAT	2457	309	1.085	19,445	702
Suspended particles less than 10 mm	PM_10_	Tons	National Emissions Inventory-SEMARNAT	2457	409	1.555	21,856	930
Ammonia	NH_3_	Tons	National Emissions Inventory-SEMARNAT	2457	338	1.018	5,699	534

As for the explanatory variables, as usual in this type of models, we include population (POP), a measure of economic activity and three variables to catch the technological components. Economic activity is represented by variable GPPC (Gross Production Per Capita), which corresponds to overall production of all economic activities in the country divided by the number of inhabitants. This variable was used as a proxy for Gross Domestic Product (GDP) because no data of the latter are available at the municipal level. In addition, GPPC and GDP are highly correlated (98.8%) and thus provide essentially the same information in statistical terms.

The proxy variables for technology have been selected based on data availability and the relation to polluting activities. The available variable that is conceptually more related to technology in the usual sense is the number of households with access to computers and the internet (HCI). These variable forms part of the Technological Achievement Index proposed by the United Nations Development Program [39], which assesses the capacity to create technology as well as the human capacity to adapt to the New Knowledge Society.

The two other variables play basically a role of control variables. The second one is the harvested area (HA), which is included to capture the polluting impact of the agricultural sector, since there is strong evidence that this sector has a significant impact on air pollution. Specifically, agricultural practices are mainly related to the emission of NO_X_, PM_10_, PM_2.5_, NH_3_ and COV [40]. Finally, we also include the per capita number of cargo and public transport vehicles (TR), because vehicles are an important source of emissions from air pollutants. Some studies analyzed the effectiveness of public policies aimed at reducing emissions, such as the restrictions to use of vehicles (see, e.g., [41, 42]). Private transport is not included in the model because it shows multicolinearity with the population variable.

**Fig 1:**
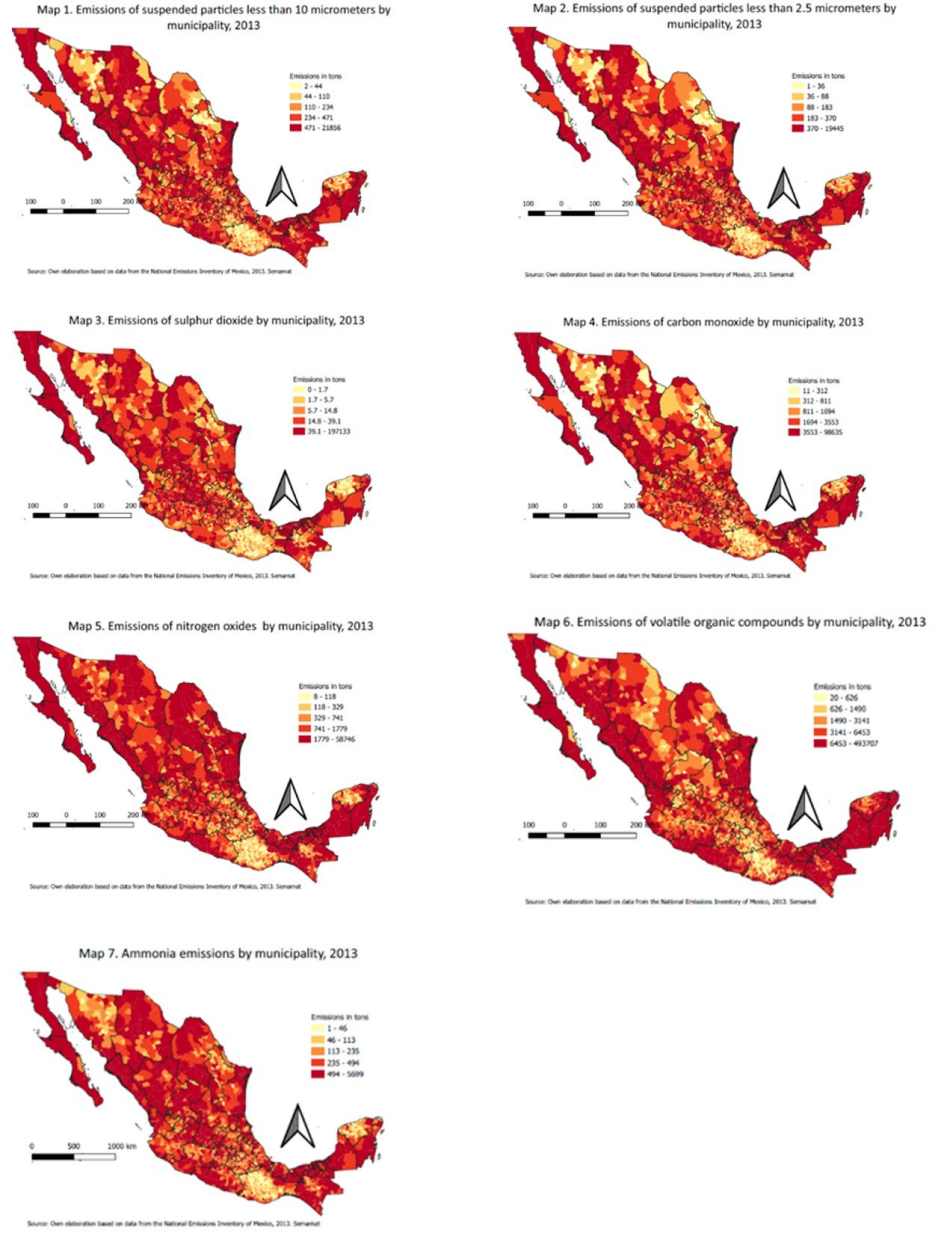
Emissions of air pollutants in Mexico.

The explained variables (POL) are the municipal emissions of seven pollutants: PM_10,_ PM_2.5,_ SO_2_, CO, NO_X_, COV and NH_3_. Fig 1 (maps 1 to 7) represent total emissions per municipality. It is worth noting that in the southern state of Oaxaca emissions of all pollutants are clearly lower than average, but for the rest of the states, the distribution of pollutants is quite heterogeneous.

The data correspond to 2013, which is the most recent year in which the relevant data are available at the municipal level. For variables whose data were not available for 2013 at the municipal level, we took the closest year available. Thus, the population variable refers to the number of inhabitants per municipality in 2010 and the number of households with access to computers and the internet refers to 2015.

### 2.3. Econometric approach. The multilevel model

Different econometric approaches have been used to estimate the STIRPAT model. These include Ordinary Least Squares [11], Generalized Least Squares [38], Robust Minimum Squares [43], Partial Least Squares [12, 44], Fixed Effects Models [29, 45], among others.

We employ a multilevel model in order to exploit the structure of our database. Multilevel models, also called hierarchical models, are specifically designed to deal with nested or hierarchically structured data [46]. In our case study there is a hierarchical structure in the sense that municipalities are nested in states. We specify seven versions (one for each pollutant under analysis) of a same multilevel model for the Mexican municipalities.

In a multilevel model there are two types of parameters: fixed (intercepts and elasticities) and random parameters (variances and covariance matrix). In each of the seven versions of our model, there are two levels. Level 1 expresses the dependent variable (amount of emissions) for observation *i* (municipality) within unit *j* (state) as:

$${LPOL}_{ij}=\beta_{0j}+\beta_{1j}{LPOP}_{ij}+\beta_{2j}{LGPPC}_{ij}+\beta_{3j}{LHCI}_{ij}+\beta_{4j}{LHA}_{ij}+\beta_{5j}{LTR}_{ij}+\varepsilon_{ij}$$ (5)

$$where\;\varepsilon_{ij}∼N(0,\sigma_{\varepsilon }^{2})$$

where *LPOL* is the natural logarithm of the volume of emissions (for every pollutant). Level 1 coefficients *β*_0*j*_…*β*_*5j*_ represent the scale term (*β*_0*j*_) and the elasticities (*β*_1*j*_…*β*_0*j*_) with respect to the explanatory variables and L*POP*, *LGPPC*, *LHCI*, *LHA*, and L*TR* represent the logarithm of the explanatory variables. Finally, *ε*_*ij*_ is the random term of level 1, which is assumed to have a normal distribution with zero mean.

In the second level of the model, each of the coefficients *β*_0*j*_…*β*_*5j*_ defined in level 1 are expressed as random variables, with a common term for all states and a stochastic term that varies across states:

$$\beta_{hj}=\beta_{h}+\mu_{hj}\;with\;h=0,1,\ldots ,5$$ (6)

For example, according to Eq (6), the level 1 coefficient *β*_0*j*_, which is the scale coefficient, can be expressed as the aggregation of two components. The first one is the systematic part, *β*_0_, which measures the average emissions of states when the explanatory variables are omitted. The second part, *μ*_0*j*_, represents the random differential component of the j^th^ state’s emissions with respect to the average value of all states. Similarly, the elasticity with respect to the population *β*_1*j*_, can be split into the average elasticity of all the states, *β*_1_, and the differential part of state *j*, which is the random tem *μ*_1*j*_. The same interpretation applies to the rest of coefficients.

By inserting Eq (6) into (5), we can rewrite the whole model as:

$${LPOL}_{ij}=\beta_{0}+\mu_{0j}+{(\beta }_{1}+\mu_{1j}){LPOB}_{ij}+{(\beta }_{2}+\mu_{2j}){LGPPC}_{ij}+{(\beta }_{3}+\mu_{3j}){LHCI}_{ij}+{(\beta }_{4}+\mu_{4j}){LHA}_{ij}+{(\beta }_{5j}+\mu_{5j}){LTR}_{ij}+\varepsilon_{ij}$$ (7)

where *β*_0_ represents the average emissions of all the states, *β*_1_…*β*_5_ is the average elasticity of emissions with respect to the corresponding explanatory variable (*LPOB*,…,LTR), *μ*_0*j*_ is the idiosyncratic term of the j-th state in terms of scale, *μ*_1*j*_..*μ*_5*j*_ are the idiosyncratic effects of the j-th state in terms of the corresponding elasticities and *ε*_*ij*_ is the level-1 error term.

### 3. Results

Eq (7) is estimated for each of the pollutants under study, with the purpose of determining the influence of the considered variables on the amount of emissions per municipality for every pollutant. The results are shown in Table 2. The estimations are carried out by maximum likelihood using STATA® software, and robustness analyses were performed to test the effects of several specifications for the random parameters. As expected, some differences were found depending on the statistical assumptions, but the internal validity of results remained for all the specifications. The obtained values of Akaike Information Criterion decrease significantly in the completely random model, which implies a better adjustment of the completely random model as compared to the null model. The “average coefficients” displayed on Table 2 are interpreted as the corresponding ecological elasticities (EE) associated to each independent variable.

**Table 2:** Multilevel model results.

*Average coefficients (Standard errors in parentheses)*							
	PM_10_	PM_2.5_	SO_2_	CO	NO_X_	COV	NH_3_
POP	0.553*** (0.026)	0.609*** (0.023)	0.857*** (0.025)	0.829*** (0. 016)	0.611*** (0.022)	0.572*** (0.03)	0.535*** (0.022)
GPPC	0.036* (0.016)	0.027 (0. 014)	0.263*** (0.034)	-0.016 (0.009)	0.078*** (0.014)	0.025 (0.02)	0.008 (0.012)
HCI	-0.168*** (0.021)	-0.198*** (0.024)	-0.032 (0.03)	-0. 138*** (0.021)	-0. 093** (0.028)	-0. 291*** (0.04)	-0. 143*** (0.023)
HA	0.357*** (0.029)	0.308*** (0.027)	0.161*** (0.021)	0.125*** (0.019)	0.241*** (0.023)	0.216*** (0.03)	0.308*** (0.024)
TR	0.029 (0.028)	0.024 (0.034)	0.438*** (0.038)	0.222*** (0.042)	0.342*** (0.033)	0.01 (0.051)	0.156*** (0.032)
Constant	-3.889*** (0.274)	-4.386*** (0.291)	-9. 501*** (0. 371)	-3. 282*** (0.287)	-3.235*** (0. 32)	-0.605 (0.424)	-3.693*** (0.241)
*Typical deviations from random parameters (Standard errors in parentheses)*							
POP	0.010** (0.004)	0.007** (0.003)	<0.01 (<0.01)	0. 002* (0.001)	0.005** (0.002)	0.013** (0.005)	0.007** (0.003)
GDP	0.002 (0.001)	0.001 (0.001)	0.018* (0.008)	<0.01 (<0.01)	0.001 (0.002)	0.004 (0.002)	<0.01*** (0. 001)
HA	0.017** (0.006)	0.014** (0.005)	0.001 (0.001)	0.007** (0.003)	0.009** (0.003)	0.016** (0.006)	0.011** (0.004)
HA	0.002 (0.003)	0.005 (0.003)	0.002 (0.003)	0.005* (0.002)	0.009* (0.004)	0.026* (0.011)	0.006* (0.002)
TR	0.003 (0.004)	0.008 (0.006)	<0.01 (<0.01)	0.028 (0.011)	0.007 (0.004)	0.033 (0.015)	0.009 (0.004)
var(Constant)	0.379* (0.247)	0.516* (0. 306)	0.072 (0.080)	1.032 (0.392)	0.901** (0.383)	2.041** (0.823)	0.220* (0.192)
var(Residual)	0.321*** (0.010)	0.311*** (0.010)	0.999*** (0.031)	0.169*** (0.006)	0.313*** (0.010)	0.387*** (0.012)	0.253*** (0.008)

* p<0.10

** p<0.05

*** p<0.001

The existing heterogeneity in emissions in municipalities can be also observed in the Table 2. Specifically, we can observe how the standard deviations of the random parameters are statistically significant at 90% level for different explanatory variables and different pollutants. This is an indicator of existing spatial heterogeneity and the adequacy of using a multilevel model. However, obtaining individual recommendations (i.e. by municipality) is something that escapes our analysis. Combining information technology and social science is an emerging research trend [47]. For this, a specific analysis should be carried out using geostatistical methods and air quality models [48], which goes beyond the objectives of this article.

In all the estimated models, the ecological elasticity of population is found to be significant, positive, and larger than the coefficients associated to the other explanatory variables. This result suggests that population is the most relevant factor for the considered pollutants in quantitative terms. Nevertheless, the ecological elasticity of population is always smaller than one, which means that the effect of an increase in population on polluting emissions is less than proportional. Across pollutants, the highest population elasticities are those corresponding to SO_2_ (0.857) and CO (0.829), which means that these pollutants are particularly sensitive to the population size. For the rest of pollutants, the corresponding elasticity is between 0.535 and 0.611.

The role of per capita production turns to be much weaker than that of population. The estimated values are well below one and, actually, they are always below 0.1 except for SO_2_, which is 0.263, meaning that a 1% increase of per capita output will result in an average increase of 0.263% of SO_2_ emissions. Apart from SO_2_, in statistical terms, the estimated elasticity of this variable is significant only for PM_1O,_ and NOx emissions.

The ecological elasticity of the proxy for technology, i.e., the proportion of households with a computer and access to the internet, is negative in all cases except for SO_2_, where it is not statistically significant at 90% level. The highest value of this parameter is found for COV with an ecological elasticity equal to 0.216. This result is consistent with the belief that technological progress can contribute to decrease environmental deterioration.

The variable related to the agricultural harvested surface area was found to be statistically significant and positive for all the pollutants, with values ranging from 0.125 (CO) to 0.357 (PM_10_). It means that pollutant emissions are increasing, but not very strongly, in the harvested area. These results are consistent with the fact that agriculture is mainly related to emissions of particulate matter, NH_3_ and NO_X._

As expected, the relative number of cargo and public transport vehicles has a positive effect on some pollutants, notably SO_2_ (with an elasticity equal to 0.438), NO_X_ (0.342) and, to a lesser extent, CO (0.222) and NH_3_ (0.156). On the other hand, the effect of this variable is insignificant in statistical terms to explain the difference across municipalities of the emissions of PM_10_, PM_2.5_, and COV. To some extent, this result may be linked to the omission of private transport from our analysis due to absence of data.

## 4. Discussion

In general terms, the studies based on the STIRPAT model conclude that energy intensity is positively linked (or, conversely, energy efficiency is negatively linked) to polluting emissions or environmental deterioration. The weight of the industrial sector is also typically found to have a positive impact on pollution, with an elasticity that varies between 0.3 and 1, whereas a larger share of the service sector on GDP is commonly associated with a lower environmental impact.

Most applications of the STIRPAT model also conclude that population and per capita economic activity have a positive effect on environmental deterioration with an observed ecological elasticity equal or close to one. [12] concluded that in middle-high income countries, where Mexico is classified, the ecological elasticity for population and income is positive but smaller than one. On the other hand, [13] concluded that for middle-income countries the population elasticity is between 1.231 and 1.907, while income elasticity ranges from 0.467 to 1.011. At the regional level, [43] estimated a cross-sectional data model for the Southeastern United States and found out that the elasticity of CO_2_ emissions with respect to population is not different from one while the results with respect to income are mixed. In a study for 20 emerging countries, including Mexico, [45] found an elasticity of 0.786 for the population and 1.121 for income.

Our empirical results about the impact of population on pollution is in line with previous studies, such as [12], who carried out an analysis in which countries are ranked by income and Mexico is considered within the medium-high income group. [45] also find a coefficient less than 1. On the other hand, in a study where Mexico is raked as a middle-income country, [13] find coefficients above 1. In a state-level sudy for the USA in 2010, [49] found similar estimated elastices than ours for CO, SO_2_ and PM_10_. Similar conclusions are also found in other studies that consider developed and developing countries together, such as [11, 28, 30, 38]. In regard to the role of the income, our results are consistent with the findings of [12], who find coefficients for the income variable with respect to CO_2_ and N_2_O emissions less than 1. In contrast, [45] find a coefficient greater than 1 for this variable. Finally, our empirical results suggest that ecological elasticity of technology can be related with the Environmental Kuznets Curve hypothesis. [50] proposed to apply the seminar study by [51], who found an inverted-U relationship between economic growth and inequality, to the relationship between economic growth and environmental degradation [52]. According to this hypothesis, in a first phase of growth there is an increase in pollution levels, up to a certain moment in which a turning point is reached and, further increases in income are associated with a decrease in pollution. If this framework is taken as valid, our results about the effect of technology can be understood as corresponding to the decreasing part of an Environmental Kuznets Curve.

## 5. Conclusions

Air pollution and its external effects is currently one of the main axes of debates in the public sphere and the policy arena. Since the Industrial Revolution, the use of fossil fuels and the increasing demand of energy have generated a global map of strong environmental degradation, with an increase in pollutant emissions that endangers ecosystems and human well-being. This is true worldwide but, especially, in developing countries. Air pollution is a serious problem in Mexico due to several socioeconomic causes, mainly through the negative effects on health and wellbeing. The emissions of local air pollutants analyzed in this study can cause, from a simple cough, to some cancers. In addition, these pollutants reduce visibility, contribute to the formation of acid rain and are precursors of smog and ozone. To shed some light on this problem, we have applied a bi-level version of the STIRPAT model to identify the main causes of air deterioration in Mexico along available data at municipal level for a set of socioeconomic variables representing the different dimensions of the model: population, per capita income, technology, harvested area and the number of cargo and public transport vehicles. Unlike most previous applications of the STIRPAT model, we have focused on local pollutants and used data at the municipal level. Using a multilevel model allows to capture the variability that exists between municipalities belonging to a state and municipalities that share characteristics of another state.

Population is found to be clearly the variable that has the greatest impact on the emissions of the 7 pollutants analyzed, followed by the agricultural harvested area. Per capita production was not significant for all models and the ecological elasticity always had a clearly lower value than one, which can be interpreted as a refutation of the traditional IPAT identity, where the elasticities are assumed to be equal to one. Our measure of technological development has a negative sign when statistically significant, which suggests that more technological advance municipalities tend, ceteris paribus, to pollute less. Finally, cargo and public transport vehicles turn out to be relevant for some pollutants, but not for all. Although population is the variable with a greater ecological elasticity, it is perhaps the one over which it is more difficult to act from a political point of view, at least in the short and the medium term. It seems more feasible to address the rest of the relevant variables to improve air quality in Mexico.

Finally, we highlight the two main limitation of this study. The first one refers to lack of updated local data. The database corresponds to 2013, which is the more recent year in which the relevant data are available at the municipal level. The second one is related to the analysis of territorial data. The STIRPAT model presents a structure defined from the economic theory. However, there are other analysis tools that would make it possible to delve into territorial differences. Mexico is not a single unit, since, as in other countries, there are areas where pollution problems are completely different, with contrast between big cities such as Mexico City, Guadalajara, or Monterrey, and cities with less socio-economic development, such as Pachuca, Querétaro, Chilpancingo, among others. New geospatial analyses can be a good complement to the STIRPAT model, providing specific territorial information for which this model is not intended.

## 6. Policymaking suggestions

Coping with the reduction of air quality and mitigating its negative effects on well-being represents a crucial challenge for national, local and regional policymakers. In order to launch effective policies and programs, it is crucial to have a good picture of the problem and, thus, a particularly relevant question is the identification and assessment of the main factors that determine the volume of polluting emissions. We did so by carrying out an analysis based on the STIRPAT model.

The fact that technological development seems relevant to mitigate environmental deterioration, together with the relevant impact of the agricultural harvested area, and the number of vehicles, suggest that an effort to improve agricultural practices and have a more efficient public transport and cargo system through technological improvements, could result in a significant improvement in air quality, life and health in Mexico. Obviously, this is a general recommendation that should be adapted to the territorial particularities and geographical restrictions. In this sense, the development of specific measures favoring the more rural territories could contribute to correct the negative externalities that urban pollutants caused on the rural environments [53]. This information can help policy-makers to improve public programs oriented to improve air quality, which is one of the challenges for the next years in Mexico and in the whole world.
